# Acute allergic rhinitis

**DOI:** 10.4102/safp.v62i1.5154

**Published:** 2020-10-12

**Authors:** Robin J. Green, Andre van Niekerk, Marinda McDonald, Raymond Friedman, Charles Feldman, Guy Richards, Fatima Mustafa

**Affiliations:** 1 Department of Paediatrics and Child Health, University of Pretoria, Pretoria, South Africa; 2 Private Practice, Netcare Clinton Clinic, Alberton, South Africa; 3 Private Practice, Blairgowrie, Johannesburg, South Africa; 4 Private Practice, Mediclinic Sandton, Johannesburg, South Africa; 5 Department of Medicine, University of the Witwatersrand, Johannesburg, South Africa

**Keywords:** allergic rhinitis, acute exacerbations, triggers, allergens, topical decongestants, intranasal steroids

## Abstract

Allergic rhinitis is a common and troubling condition. Basic management of this condition has been well described. However, acute exacerbations of the chronic condition allergic rhinitis are a seldom discussed or described problem despite the fact that even well-controlled patients frequently have exacerbations. This consideration means that a new approach is necessary to define the management of these patients. There are three important events that illustrate the need for a new therapeutic approach:

- A person who gets a new diagnosis of allergic rhinitis, but has symptoms for many months or years
- A sufferer of allergic rhinitis who is exposed to an environment that triggers an exacerbation
- A person who has an exacerbation related to another trigger.

Recognition of triggers and management strategies to correctly use ‘relief’ therapies such as topical nasal decongestants is the key to successful management. In addition, the use of an ‘action plan’, as for asthma, is useful.

## Introduction

Allergic rhinitis (AR) is a common inflammatory process that manifests as nasal itchiness and congestion, postnasal drip, sore throat, cough, itchy swollen eyes and sometimes airway hyper-reactivity (AHR) if asthma coexists. It is associated with considerable disability.^1,2,3^

**FIGURE 1:**
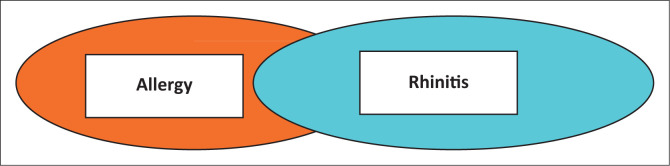
Allergic rhinitis = allergy + rhinitis (inflammation of the lining of the nose).

**FIGURE 2:**
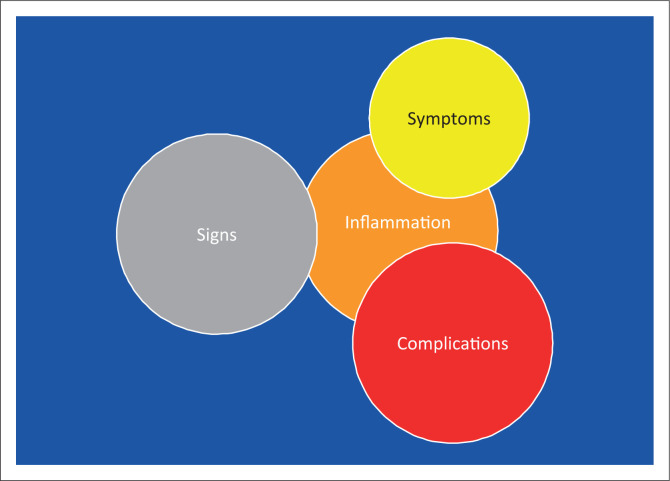
Inflammation determines clinical problems in the nose.

The term allergic rhinitis suggests two factors which operate together to produce disease. They are allergy and rhinitis (Figure 1). Allergy is a special type of hypersensitivity response in which the primary immunological mechanism, in the case of the nose, is usually immunoglobulin E (IgE)-mediated. Rhinitis is defined as inflammation of the lining of the nose. Both factors need our attention in nasal disease, and only by a joint approach, is successful therapy possible. Allergy defines the pathogenesis of the problem, whereas inflammation results in the clinical problem (Figure 2). A joint therapeutic approach is fundamental to a holistic solution.

Acute exacerbations of the chronic AR are a seldom discussed or described problem despite the fact that even well-controlled patients frequently have exacerbations. This consideration means that a new approach is necessary to define the management of these patients. There are three important events that illustrate the need for a new therapeutic approach:

1. A person who gets a new diagnosis of AR but has symptoms for many months or years.
2. A sufferer of AR who is exposed to an environment that triggers an exacerbation.
3. A person who has an exacerbation related to another trigger.

## Recognising acute rhinitis in adults and children

Acute rhinitis, allergic and/or non-allergic, is common and will affect all people at some time. The onset is abrupt and usually lasts for a couple of days. Children are especially vulnerable and episodes occur up to four times more frequently when compared with adults. Events are often perceived as trivial and self-limiting but may become very troublesome. It is particularly important to recognise and manage them appropriately when acute exacerbations are due to allergy.

The history of the presenting symptoms and a past personal history are often more helpful than a clinical examination. A proper history will offer the first step towards an accurate diagnosis. The inception of allergic rhinitis may require up to two seasons of allergen exposure before a significant clinical presentation manifests.^4^ Children who are younger than 2 years of age will often be thought to have a viral infection (a common cold) rather than acute AR. A patient with a personal previous history of allergic disease (such as atopic dermatitis, allergic asthma and allergic conjunctivitis) will be more prone to AR, whilst systemic complaints (such as fever or a sore throat) would suggest an acute viral aetiology, and the symptoms associated with viral rhinitis also last longer in children than in adults.

Acute AR is usually associated with paroxysms of sneezing, anterior and posterior rhinorrhoea, nasal obstruction and nasal itch. The complaint of ‘itch’ is often prominent in acute allergic rhinitis and patients may also report itching of the palate and eyes (sometimes with tears). Troublesome symptoms such as sleep-disrupting breathing and impairment of daily activities will be noted in more severe disease. Children are often not efficient at nasal clearance and parents may be frustrated by snorting, sniffing and tongue clicking associated with an itchy palate.

The next step is to pay attention to external clinical signs and to perform an anterior rhinoscopy, otoscopy and throat examination. Allergic shiners, Dennie–Morgan lines, a transverse nasal crease, allergic facies and allergic mannerisms will support a diagnosis of AR. Swelling of the nasal mucosa, the presence of clear secretions and a pale or pale-bluish colour to the nasal mucosa will further support an allergic aetiology. A beefy red mucosa is often seen with viral infections. The presence of serous middle-ear effusions, retracted tympanic membranes and lymphoid hyperplasia (cobbling) of the posterior pharynx may also suggest an acute exacerbation of AR.

It remains paramount to demonstrate the presence of allergen-specific IgE (e.g. through allergy skin prick testing) before a diagnosis of AR is made. Only a subset of sensitised individuals will demonstrate clinical allergy, and it is valuable to seek correlation between allergen exposure and the presence of symptoms.

## Specific trigger factors

Acute rhinitis triggers can be allergic or non-allergic. Non-allergic triggers include infections, medication or occupational factors such as exposure to irritants such as wheat in mills or when baking, which can lead to symptoms of both rhinitis and asthma. Vasomotor and idiopathic rhinitis are more difficult to both diagnose and treat.

Allergic triggers can be differentiated by the duration of the symptoms: whether perennial (year-round) or seasonal or whether there is an association with being indoors or outdoors (Table 1). Indoor environmental allergens that are perennial are house dust mite, and cat and dog dander. House dust mite allergy tends to be worse at the coast as opposed to the dryer Highveld as mites need ambient humidity to procreate and grow. However, what has emerged is that even perennial indoor allergens can cause exacerbations of rhinitis when individuals become house-bound as has occurred during the coronavirus disease 2019 (COVID-19) ‘lockdown’. In addition, in winter periods, when indoor living coincides with winter bedding taken out of storage, exacerbations of AR may occur even in mid-winter, outside of the pollen season.

**TABLE 1:** Distinguishing acute allergic rhinitis symptoms.

Type	Aetiology
**Acute rhinitis**	
Viral infection	Most likely viral infection if symptoms less than 2 weeks
Seasonal rhinitis	Correlates with outdoor allergens such as grass, tree and weed pollen, and *Alternaria and Cladosporium* (mould) www.pollencount.co.za
Pollution spike	Increased indoor pollution: prolonged time spent indoors, fires, gas stoves, pets, air conditioner filters Increased outdoor pollution https://aqicn.org/country/south-africa
**Chronic rhinitis**	
Perennial allergens	Outdoor allergens: grass, mould www.pollencount.co.za Indoor allergens: house dust mite, cat, dog, mould
Non-allergic perennial triggers	Outdoor or indoor pollution https://aqicn.org/country/south-africa
Chronic rhinosinusitis	Sinus infection that lasts more than 6 weeks Consider immune deficiency
Review if recurrent	Is it the correct diagnosis? Is there adequate adherence to medication?

The primary outdoor perennial allergen in Gauteng, Limpopo, North West and Free State is grass, as grass pollen is present for at least 10 months of the year in these areas.^5^ Wild indigenous grasses such as Buffalo (thatch grass) can be tested for by Zea Maize skin prick test and serum IgE as it is part of the same family. Rye grass grows wild in the temperate areas of South Africa such as the Western Cape and Kwazulu-Natal, where it is used as a pasture grass and can be cultivated in areas with < 780 mm rain per year. Bermuda grass is a perennial outdoor allergen that cross reacts with Kikuyu and Eragostis grasses.^5,6,7^

Mould allergens, *Aspergillus, Alternaria* and *Cladosporium,* are present for 10 months of the year as outdoor allergens in Gauteng and in coastal areas, especially in more humid conditions and with temperatures of between 18 °C and 30 °C. Damp and poorly ventilated houses may be a major contributor to AR in poorly ventilated houses, especially if damp is present.

Seasonal allergens tend to be more prevalent in outdoor environments and can be acute as the season begins. These include moulds such as *Alternaria, Cladosporium* and *Aspergillus*, as well as pollens from trees and weeds such as Khakibos. These recommendations have been drawn from pollen monitoring in the 1990s but following the new pollen monitoring process that commenced in August 2019 (www.pollencount.co.za), it seems that previously pure seasonal allergens such as *Alternaria* and *Cladosporium* and tree pollens are persisting for longer. Advising patients to monitor symptoms with pollen counts can give valuable information to guide therapeutic advice.

### Non-allergic triggers

Non-allergic triggers such as irritants (cleaning agents and perfume) can often be clearly pointed out by the patient. Indoor pollution can act as an irritant causing rhinitis itself or as an exacerbator of AR. Symptoms of vasomotor rhinitis overlap with those of AR but are not caused by an immune reaction. It is however critical that allergies first be ruled out.

As many as 11 acute infections can occur in children per year; however, these are usually self-limiting and need little in the way of medication. Acute rhinosinusitis lasts < 2 weeks, whereas chronic rhinosinusitis may last more than 6 weeks and can be mistaken for AR, given the prolonged nature of the symptoms. All three of these conditions can occur simultaneously but a good history can help to distinguish them.

Recurrence of acute rhinitis or recurrent complaints about chronic rhinitis need to trigger an evaluation of the diagnosis, medication used and medication technique. Patient adherence is often a problem. The latter may require obtaining insight into a patient’s knowledge of their condition, specifically whether they are aware which medications are for chronic use and which are for acute exacerbations.

## Clinical examination and special investigations required

It is critical that any adult or child who is known to have AR and who then develops acute symptoms should be carefully assessed.^8^

**FIGURE 3:**
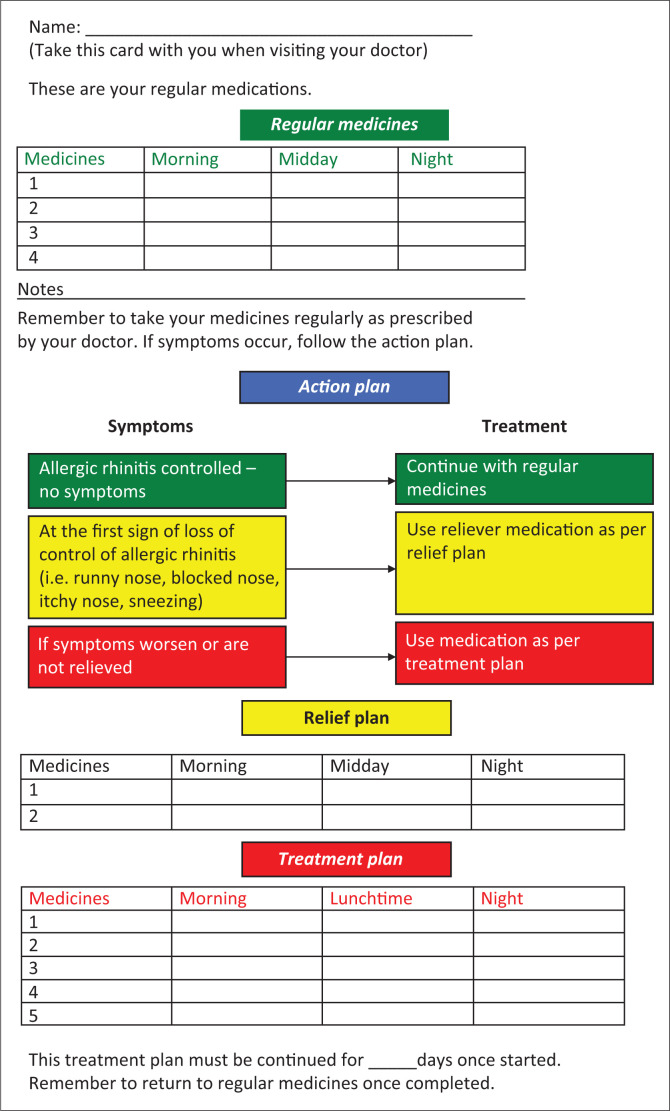
Patient directed management card for allergic rhinitis.

Mild exacerbations, and those who are well educated as regards their condition, may be self-managed according to an action plan (see Figure 3). However, a patient who has more severe symptoms or has no prior health contact requires a careful history to elicit symptoms and triggers.

If an examination is required, it should include the entire upper respiratory tract (nose, ears and throat), as well as the face for signs of an allergic facies.^8^

Special investigations, such as allergy testing, may be required if an allergen that is avoidable is suspected. However, testing for viruses, bacteria and other microorganisms from respiratory tract secretions is not appropriate for isolated upper respiratory tract symptoms.^9^

A computed tomography scanning of sinuses is seldom required, even when acute rhinosinusitis is suspected.^10^

## Management using topical decongestants including anti-inflammatory strategies for oxymetazoline

Pharmacotherapy is the mainstay of treatment for AR in patients with symptoms of nasal allergy varying from mild to severe, and a number of potentially effective medications are available for both relief of episodic acute symptoms and the prevention of chronic symptoms.^11^

Amongst the treatments are the topical decongestants, which are alpha-adrenergic agonists that cause vasoconstriction, and thus reduce mucosal oedema and therefore nasal congestion.^11^ The active agents in topical decongestants are either catecholamines (e.g. phenylephrine) or imidazolines (e.g. oxymetazoline and xylometazoline) and are classed as vasoconstrictor sympathomimetic agents.^12^ These agents have been indicated for use in vasomotor rhinitis and as add-on therapy for AR, as well as for the treatment of viral illness, sinusitis, otitis media and eustachian tube dysfunction.^13^ Topical decongestants are effective at reducing nasal congestion rapidly, and therefore nasal patency, but they do not affect other AR symptoms such as nasal itching, rhinorrhoea or sneezing.^12,14^

Nasal decongestants have limitations for long-term use, but they are useful for short-term use until either the underlying inflammatory process resolves or another appropriate long-term therapy, such as intranasal corticosteroids, becomes effective.^11^ One of the potential complications of prolonged use of topical decongestants is rhinitis medicamentosa (RM), a condition in which there is a rebound symptomatic vasodilatation and nasal congestion.^14^

Studies attempting to determine the duration of topical decongestant use that leads to RM are unhelpful, as RM developed in the different studies after varying amounts of time.^14^ A recent publication by the same authors as this article recommended that topical decongestants should, as a rule, be used for a maximum of 7–10 days.^8^

With regard to the imidazoline derivatives, one study over 18 days indicated that nasal zylometazoline was a stronger decongestant than nasal corticosteroids (mometasone furoate).^15^ Another study documented that combining mometasone furoate and oxymetazoline relieved seasonal AR symptoms, including congestion, faster than the intranasal steroid alone.^16^ Furthermore, several other studies reported that the combination of intranasal steroid and intranasal oxymetazoline was more effective in controlling AR symptoms than either agent alone.^17^

In addition, and possibly contributing to its efficacy at reducing AR symptoms, oxymetazoline has been shown to exhibit antioxidative and anti-inflammatory properties *in vitro*.^18,19,20,21,22^ Amongst these effects are inhibition of pro-inflammatory reactions involving arachidonic acid-derived metabolites,^18^ modulation of pro-inflammatory cytokines and of the T-cell stimulatory capacity of dendritic cells,^19^ and inhibiting and resolving inflammatory reactions in human neutrophils.^20^ Additional studies have documented that oxymetazoline has potent antiviral activities particularly with regard to the rhinovirus.^21,22^

### Other management strategies

Management should be directed to the nose primarily but extra-nasal and systemic symptoms should receive attention as well. The aim of therapy would be to relieve the symptoms as rapidly as possible, which requires administration of appropriate pharmacotherapy rather than just allergen avoidance, which, although an important overall component of therapy, is more relevant to chronic management rather than the acute episode.

### Pharmacotherapy

Treatment consists primarily of administration of anti-inflammatory (or controller therapies) along with symptomatic (or reliever) therapy, the latter of which consists primarily of decongestants and antihistamines (AH).

### Corticosteroids

Whereas intranasal corticosteroids (INCS) are the mainstay of therapy, on rare occasions a short course of oral corticosteroid (OCS) might be necessary.^23,24^ This is, however, an uncommon situation and would be dependent on comorbidity such as asthma or nasal polyps, if symptoms are severe or INCS are not tolerated. The dose would be with a prednisone equivalent of 30 mg daily × 3–5 days. Oral corticosteroid if utilised should be administered along with INCS of which there are many different proprietary intranasal products that utilise a variety of corticosteroid preparations. These include beclomethasone, budesonide, fluticasone propionate and furoate, ciclesonide, mometasone and triamcinolone. Some are administered once daily and some twice daily depending on the potency and affinity for the glucocorticosteroid receptor.^25^

Intranasal corticosteroids are superior to AH; and leukotriene receptor antagonists in relieving all the symptoms associated with acute AR including ocular symptoms and AHR if AR and asthma coexist. Antihistamines do have a faster onset of action.^26^

### Antihistamines

These agents have a relatively fast onset of action and are useful as adjunctive therapy to INCS. They are available as first- and second-generation H1 receptor blockers. They are effective at reducing symptoms such as nasal itchiness and ocular symptoms but are less effective at relieving nasal congestion. The second-generation AH are less lipid soluble and as such cross the blood–brain barrier with difficulty obviating the sedating and cognitive effects of the first-generation agents.^26^

The agents that are available in South Africa are cetirizine, loratadine, desloratadine, fexofenadine and levocetirizine. Rupatidine is an agent that has both antihistaminic properties and being a PAF antagonist. It is non-sedating and inhibits the degranulation of mast cells and the release of TNF-α from mast cells and monocytes.^27,28^

A topical AH such as azelastine nasal spray or olopatadine in combination with an INS, may be of value if the response to an oral therapy is inadequate.^29^

### Leukotriene receptor antagonists

The leukotriene receptor antagonists (LTRAs) are effective therapies in some patients who have an inadequate response to AH. As monotherapy, a meta-analysis suggested that they may be more effective in alleviating nocturnal (difficulty going to sleep, night-time awakenings and nasal congestion on awakening) more than daytime symptoms (congestion, rhinorrhoea, pruritus and sneezing).^30^

When used in combination there are added benefits specifically in those with perennial rhinitis for the control of symptoms of rhinoconjunctivitis, specifically a composite of nasal symptoms, rhinorrhoea and sneezing.^31^ A recent ‘black-box’ warning has warned of psychological side effects that may occur in some patients and patients should be observed for depression or other symptoms.

In asthma, there is a heterogeneous response to the LTRA that appears to be related to genetics.^32^ This is probably also the case with AR, and if no benefit accrues after 2–4 weeks, they should be stopped.

### Antibiotics

Antibiotics have no role in the management of acute AR, even if the exacerbation is thought to have an infectious aetiology.

## Acute rhinitis and coronavirus disease 2019

COVID-19 is caused by the severe acute respiratory syndrome coronavirus 2 virus (SARS-CoV-2). It was first detected in China in late 2019 and the infection has now been declared a pandemic by the World Health Organisation (WHO).^33^ The presentation of the infection ranges from asymptomatic to a severe acute respiratory syndrome. Most people, however, present with mild disease, the symptoms of which include fever, upper respiratory tract ‘cold’-like symptoms, cough and shortness of breath.

Studies, mostly from China, reveal that patients with common allergic diseases do not develop unusual or more severe symptoms.^33^ Only individuals who had chronic obstructive pulmonary disease (COPD) were at a greater risk to develop a secondary bacterial pneumonia and were more likely to progress to severe disease.

### Spread of the coronavirus

The virus is spread in respiratory droplets through coughing or sneezing. Droplets may reach as far as 1 m – 2 m from the person sneezing or coughing. It is recommended that everyone should adopt cough/sneezing etiquettes, that is, to cough/sneeze into the elbow or a tissue and throw it away and thereafter thoroughly wash hands. Sick people and healthcare practitioners should wear a mask to help prevent the spread of the virus.

The coronavirus may survive in the air for up to 8 h and on surfaces for up to 6 days.^34^ This may be the single most important reason to ensure stringent control of acute rhinitis.

*The most important advice to asthmatics to protect themselves is to keep their asthma under control and that includes controlling AR.*

### Allergic rhinitis in a time of Covid-19

There has been no evidence to suggest that people with AR have an increased risk to become infected with the coronavirus relative to others. However, they should practice caution and remain safe.

Individuals with AR should strictly comply with their regular treatment regimens, especially INCS as there is undisputed benefit related to their use^35^ and importantly they do not appear to increase the risk of getting the coronavirus. It is imperative to distinguish that nasal symptoms are because of AR and not coronavirus; therefore, if upper respiratory tract symptoms such as coughing, sneezing and shortness of breath develop, a doctor should be consulted.

Asthmatics with eczematous skin should seek advice on skin protection because of frequent use of corrosive disinfectants and increased frequency of hand washing.

## An action plan for acute rhinitis

As acute symptoms of allergic rhinitis may arise for the reasons explained above and often do so suddenly and unpredictably, we suggest that every patient who receives a diagnosis of AR should receive an ‘action plan’ to manage his or her condition appropriately and safely (Figure 3).

## Conclusion

Acute exacerbations of allergic rhinitis affect many individuals with the disease, even those who are apparently well controlled. Triggers of exacerbations include both new allergen exposures and non-allergic triggers. Recognition of symptoms and their associated impact is critical in considering acute therapies. Interventions for acute symptoms include topical nasal decongestants, antihistamines and intranasal corticosteroids. It is useful to provide sufferers of allergic rhinitis with an ‘action plan’ to pre-empt acute symptoms and suggest therapies to be used, even before consulting a healthcare professional.
